# Extensive high-pressure injection injury of the hand due to epoxy resin paint: a case report

**DOI:** 10.1080/23320885.2021.1898965

**Published:** 2021-03-15

**Authors:** Gaku Niitsuma, Hidechika Nakashima, Takushi Nagai, Kenichirou Teramoto, Keikichi Kawasaki, Katsunori Inagaki

**Affiliations:** a Department of Orthopaedic Surgery, Showa University School of Medicine, Tokyo, Japan; b Kumamoto Kinoh Hospital, Kumamoto, Japan; c Showa University Northern Yokohama Hospital, Kanagawa, Japan

**Keywords:** High-pressure injection injury, epoxy resin, hand surgery, pedicled groin flap

## Abstract

Epoxy resin paint has rarely been inoculated in high-pressure injection injuries. We report the surgical management of a patient with a rare extensive high-pressure injection injury caused by epoxy resin paint. A pedicled groin flap was used, as it prevents tissue scarring, tendon adhesion, and contracture of the fingers.

## Introduction

High-pressure injection injury is a puncture wound caused by a broken paint gun or high-pressure hose, followed by the injection of water, grease, paint, gasoline, oil, or thinner. This injury is relatively rare among various hand injuries. Epoxy resin paint is known to cause occupational contact dermatitis. However, it has rarely been inoculated in high-pressure injection injuries. Two large-scale reports on high-pressure injection injuries were found; the first, from Japan, comprised 89 cases [1], and the second was a systematic review of 49 articles, comprising 115 cases, conducted by Eells et al. [2]. However, neither report contained details about epoxy resin paint-induced injury. Valentino et al. [3] described four cases of epoxy resin paint-induced injury among 12 cases of high-pressure injection injuries from paint guns in shipbuilders. Thus, there is a dearth of literature on the intraoperative findings or specific treatments for such an injury, especially for one as extensive as that experienced by our patient. Most reports included injuries to a single finger only, but the injury in our patient extended from the palm to the tips of several fingers. The prognosis depends on the type of injected substance and the region affected. In comparison to other substances, epoxy resin paint has a unique property in that it hardens within 24 h. This necessitates immediate surgical debridement.

Herein, we report the surgical management of a patient with a rare extensive high-pressure injection injury of the hand caused by epoxy resin paint. Informed consent (permission) was obtained from the patient to publish his clinical photographs, and the protocols of this case report conform to the principles laid down in the Declaration of Helsinki. Although this report was not approved by an institutional review board, the patient identity is not harmed, as all data are anonymized, and the personal information and privacy of the patient are protected in this study. We also agreed to not use other data collected from the patient that were not specific to this report to minimize time constraints and to enable the patient to easily withdraw these data, even after consenting.

## Case report

A 33-year-old man, employed in the shipbuilding industry, was brought to the hospital with pain his left palm and fingers, along with disturbances in sensation. An accidental injury had occurred when a deteriorated hose used for spraying paint under high pressure had broken. This led to a high-pressure injection of paint through the wound in his hand. He was urgently transferred to our hospital and admitted for surgery on the day of injury.

**Figure 1.**
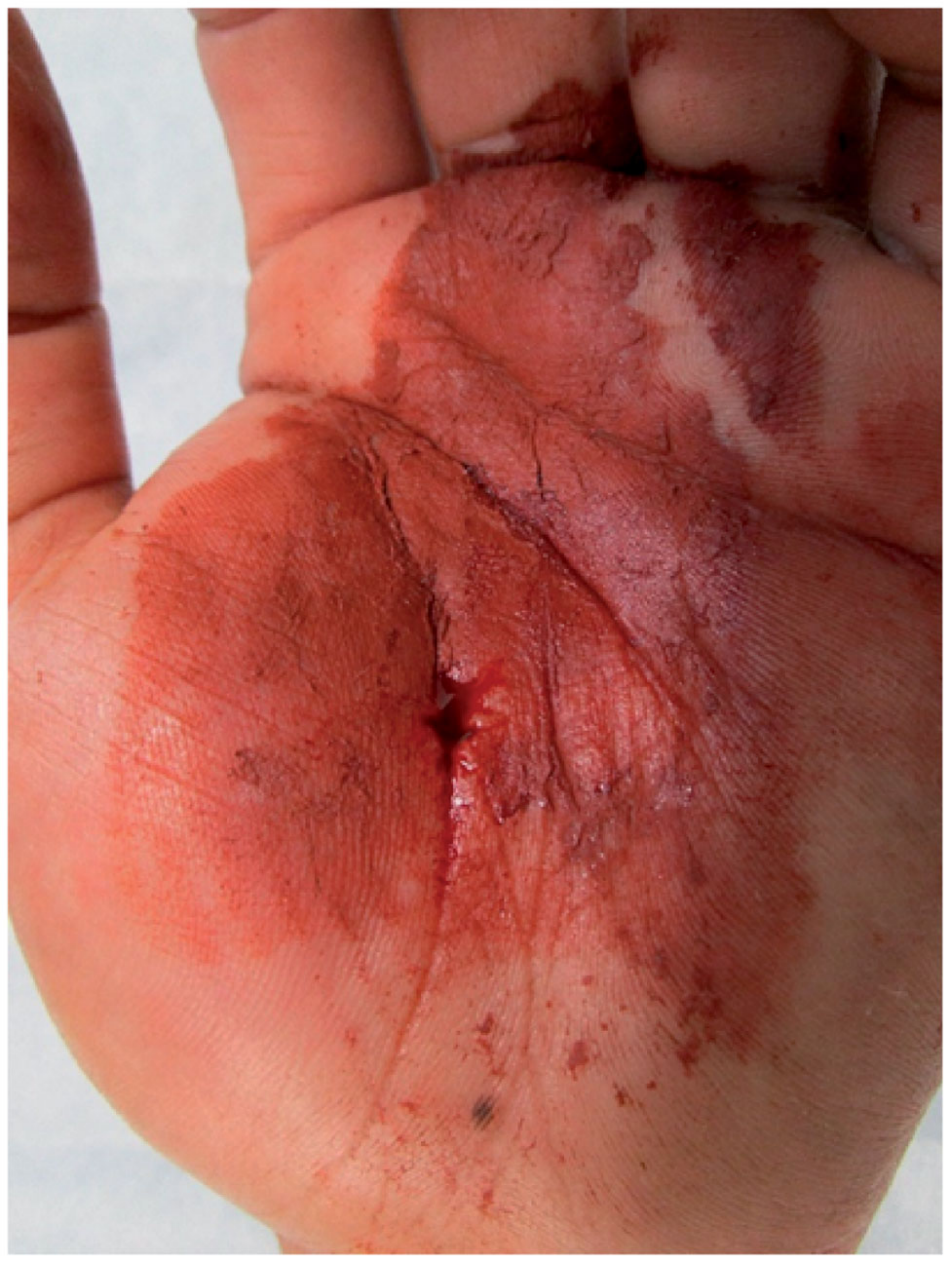
A small wound is observed on the thenar aspect of the thumb, and the paint is attached to the surrounding wound.

On examination, the right hand was determined to be dominant; thus, the injury was on the non-dominant side. An entrance wound approximately 1 cm in length was found in the middle of the thenar eminence. Reddish-brown paint spray was noticed around the wound (Figure 1). There was severe tenderness and swelling in the left hand, with restriction of active movement in all fingers. There was no sensation in any finger. However, the capillary refilling time was normal. There was no pain in the carpal tunnel, wrist joint, or forearm.

**Figure 2.**
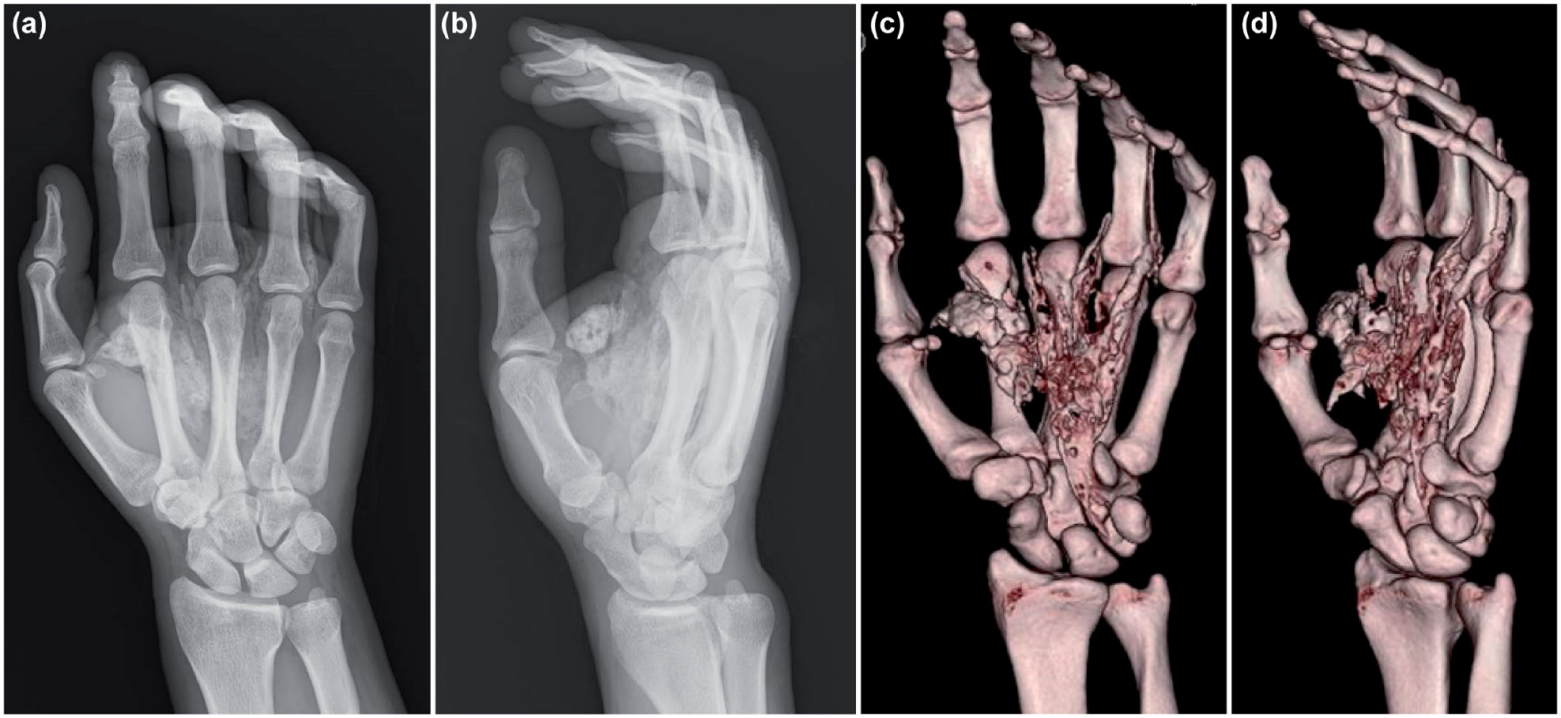
X-ray (a, b) and computed tomography (c, d) images show that the paint spread extensively along the flexor tendons of the palm.

There were no abnormal findings in the blood, chest X-ray, and electrocardiography examinations. X-ray and computed tomography (CT) images of the hand showed extensive paint inoculation, extending from the carpal tunnel proximally, to the index, middle, and ring fingers distally, directed along the digital neurovascular bundles (Figure 2(a–d)). In the ring finger, paint was observed along the extensor tendon, extending from the metacarpophalangeal (MP) joint to the dorsal side of the proximal phalanx. There were no fractures noted.

**Figure 3.**
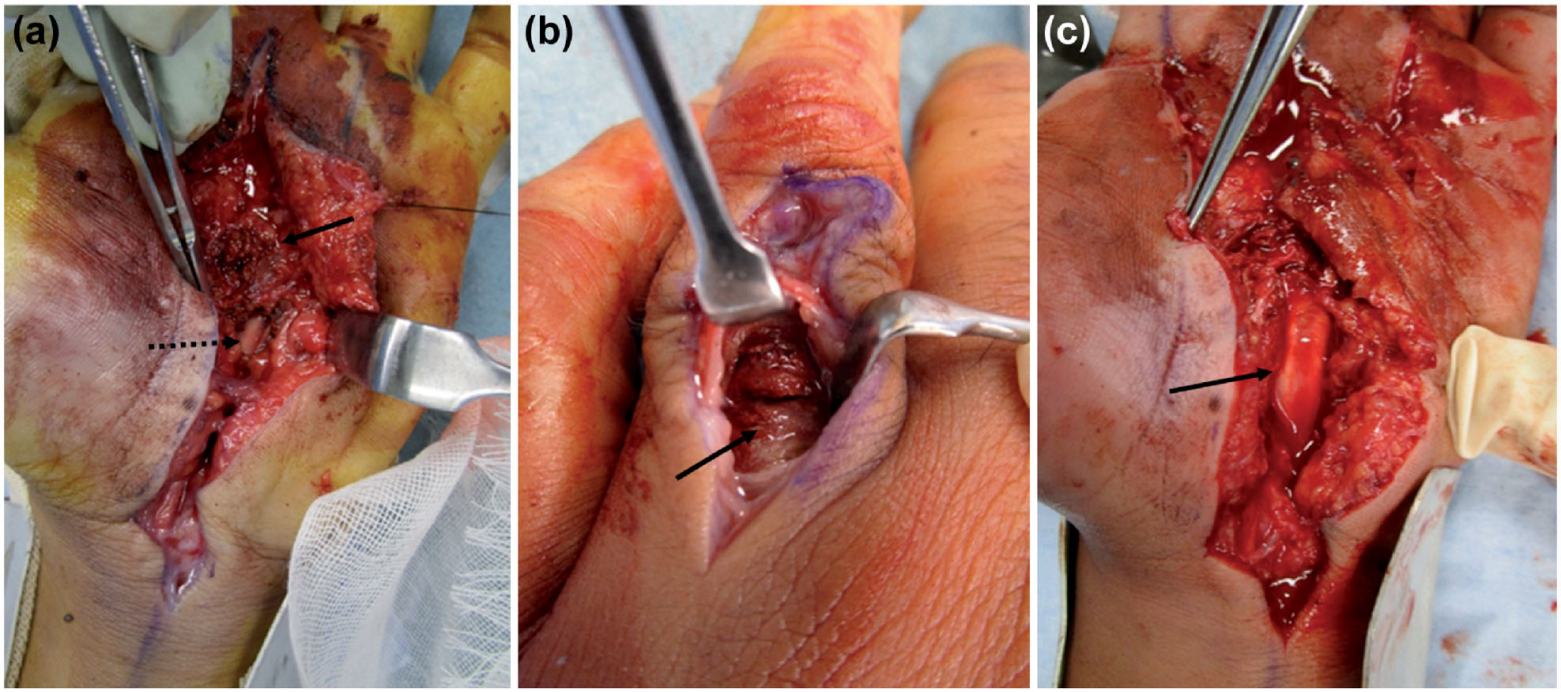
(a) Paint is observed inside the carpal tunnel (arrow: paint, dashed arrow: median nerve). (b) There is also paint on the dorsal side of the proximal phalanx (arrow). (c) After removing paint, the median nerve is evaluated (arrow: median nerve).

The patient underwent emergency debridement following an axillary nerve block. A Bruner’s zig-zag skin incision was made from the MP joints of the index and middle fingers to the carpal tunnel. The skin incision was then extended to include the MP joint of the ring finger. The reddish-brown paint had penetrated the palmar aponeurosis and entered the carpal tunnel. We could not completely remove it because the paint had a consistency like ‘chewing gum’. When the carpal tunnel was incised from the normal side at the proximal end, the internal pressure was noted to be high, and the paint exuded out from the distal end (Figure 3(a)). The inner recesses of the carpal tunnel were clear, and there was no paint on the proximal carpal bones.

Incisions distal from the carpal tunnel revealed paint on the first annular pulley of the index, middle, and ring fingers. The paint had not adhered to the flexor tendon and could be removed. It had not reached the volar side of the proximal phalanx of the index and middle fingers. In the ring finger, paint was found on the dorso-distal end of the proximal phalanx, alongside the digital arteries from the MP joint. A longitudinal incision was made on the dorsal side of the proximal phalanx of the ring finger, and paint was noted on the extensor tendon synovium (Figure 3(b)). We performed debridement from the volar to the dorsal side of the ring finger. In the MP joints of the index, middle, and ring fingers, the paint was adherent to the neurovascular bundles. We attempted to debride it completely (Figure 3(c)), but the paint had tracked around the neurovascular bundles and could not be removed. In addition, paint was observed on the tendon sheath, but it had not adhered and could be easily removed. However, at the site of the injection, the paint was adhered around the palmar aponeurosis, subcutaneous soft tissue, and neurovascular bundles of the fingers. A suction drain was placed in the carpal tunnel, and the incisions were partially closed. A dorsal splint was applied on the hand.

**Figure 4.**
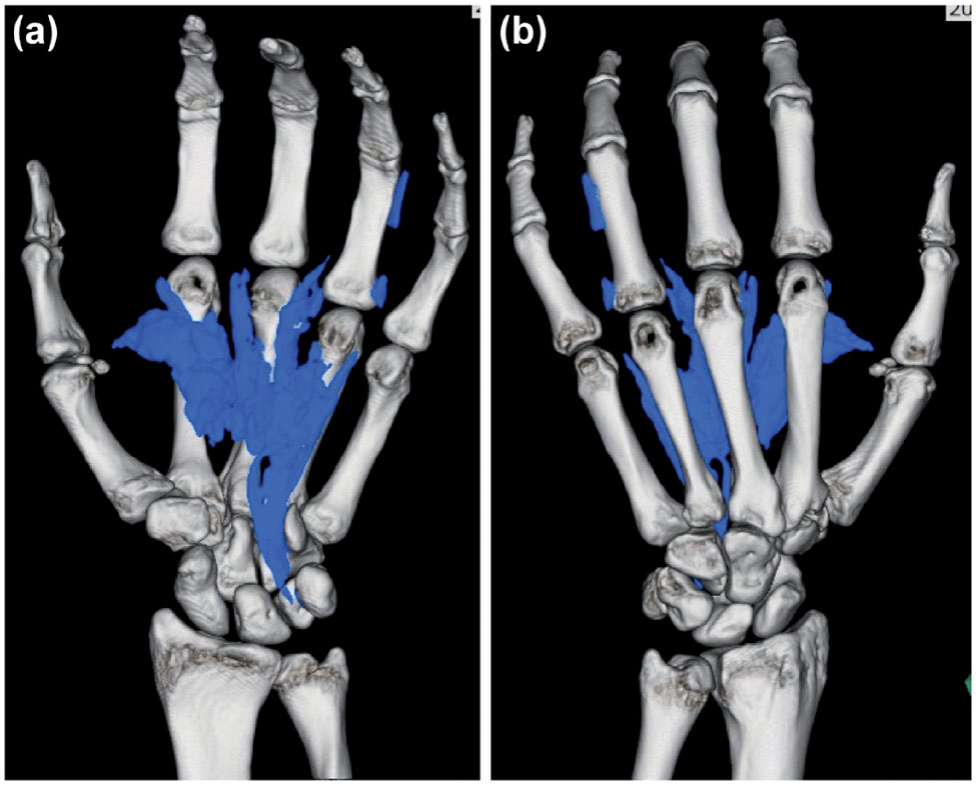
Computed tomography images acquired after the first operation show paint in the thenar space and the index, middle, and ring fingers.

**Figure 5.**
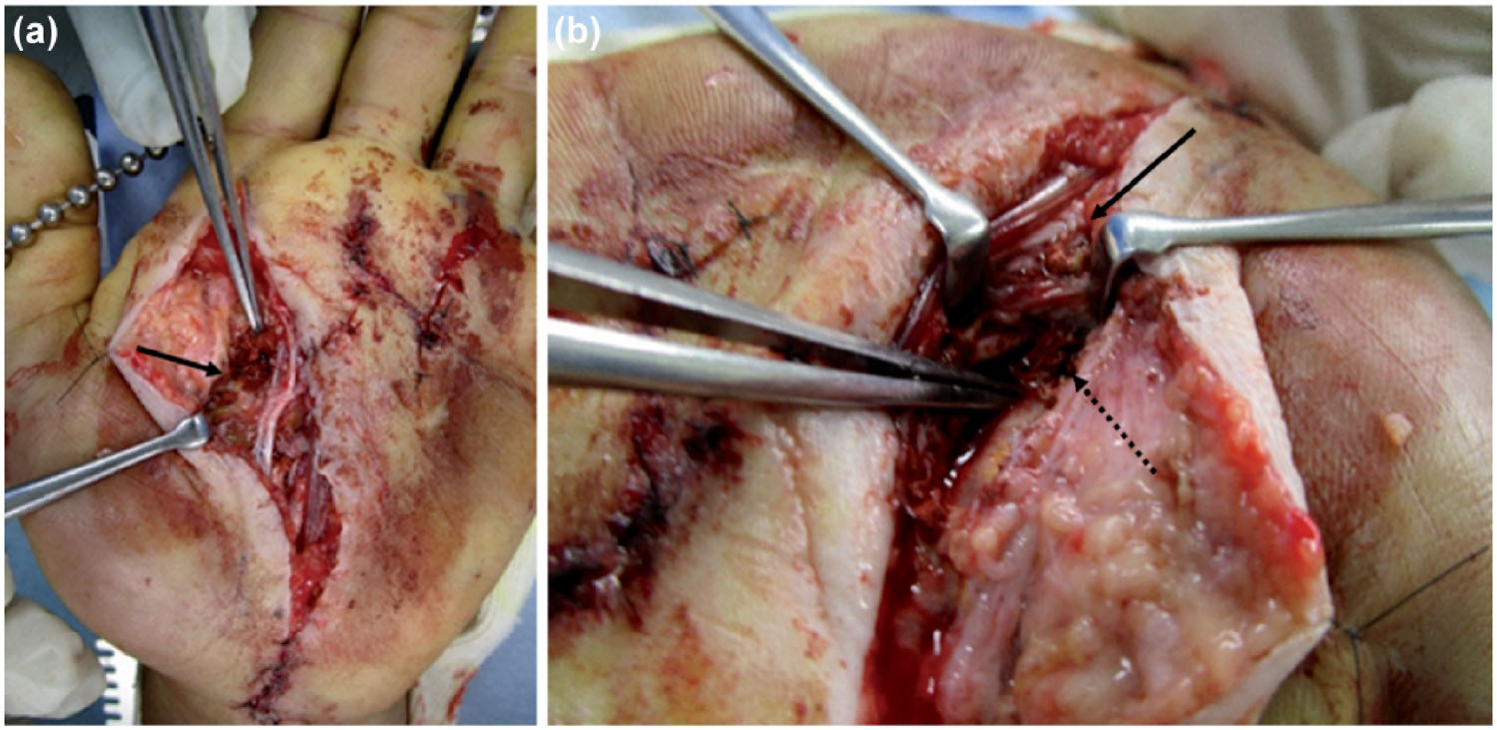
(a) Clay-like adherent paint is observed in the thenar space (arrow). (b) After removing paint, the inside of the carpal tunnel was checked, and paint is observed between the flexor tendons (solid arrow: palmer aponeurosis, dashed arrow: paint).

On the first postoperative day, numbness and pain in the fingers had not decreased. Hence, repeat emergency CT was performed, and the retention of additional paint residue was noted, which was more than that anticipated (Figure 4(a,b)). We had to re-perform emergency debridement under axillary nerve block. A skin incision was made from the MP joint of the index finger to the carpal tunnel (as in the first surgery). Hardened-block paint was detected in the subcutaneous tissue, on the palmar aponeurosis, and above the tendon sheaths (Figure 5(a)). In addition, adherent paint was found in the thenar space and around the neurovascular bundles of the fingers. The paint remnants in the thenar space and carpal tunnel had tracked from the injection site (Figure 5(b)). The palmar aponeurosis was excised, and debridement was performed. A suction drain was placed in the carpal tunnel. A dorsal splint was re-applied, which enabled the active range of motion (ROM) of the fingers. The patient received antibiotics intravenously for 1 week (ceftriaxone 2 g/day) after the first and second surgeries.

On the first day after the re-operation, the drain was removed, and rehabilitation of the fingers was initiated to facilitate the active ROM. Subsequently, although numbness remained, sensation to the area of sensory innervation of the median nerve had significantly improved. On the third day, passive ROM exercises of the fingers were started, and a dynamic splint (flexion rubber band, outrigger splint, and Kleinert splint) was employed. The ROM improved to normal. However, the skin necrosis that remained in the center of the palm around the injection site was extensive, and flap coverage was planned.

**Figure 6.**
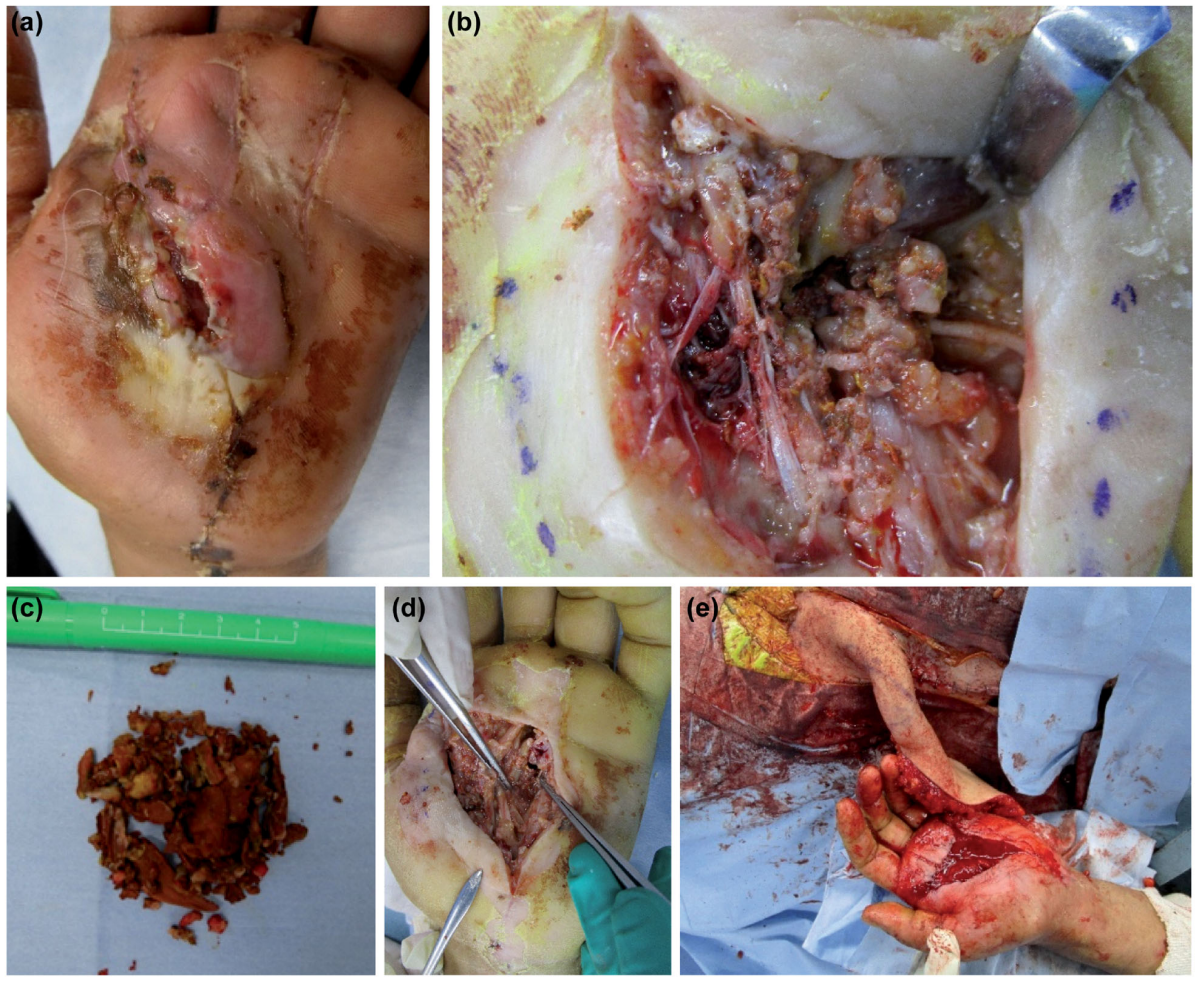
(a) Extensive necrosis is observed around the wound. (b) Debridement of the necrotic epidermis reveals residual paint in the carpal tunnel. (c) The extracted paint is shown. A large amount of cured paint was also observed during the third operation. (d) After debridement, a fan-shaped skin defect is seen in the hand. (e) Reconstruction was performed with a pedicled groin flap.

We waited 3 weeks after the re-operation to identify the necrotic area (Figure 6(a)). Debridement was performed a third time under general anesthesia. The dorsal aspect of the flexor tendons in all fingers was inspected, and a large amount of paint was found on the dorsal side of the flexor digitorum profundus (Figure 6(b)). We performed debridement again to remove the paint. The paint had become easier to remove because it had hardened completely and could be separated from the soft tissues (Figure 6(c)). The necrotic epidermis was debrided from the thenar to the MP joints of the index, middle, and ring fingers. An extensive fan-shaped skin defect remained (Figure 6(d)). Since the defect on the palm was widespread, reconstruction was performed using a pedicled groin flap (Figure 6(e)). The ROM of the fingers was improved, and the pain, numbness, and paresthesia were reduced. Five weeks after the first operation, the flap was divided, and the patient was discharged 3 weeks later.

**Figure 7.**
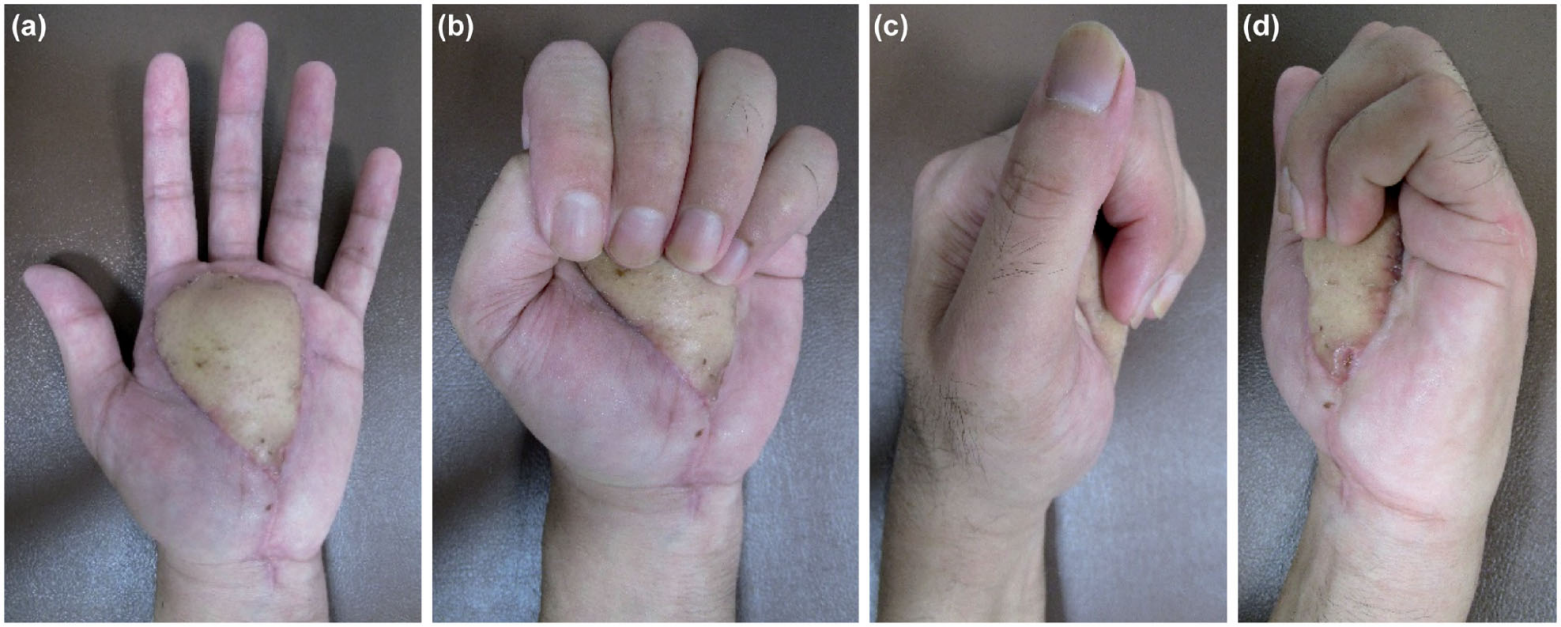
(a–d) No limitation in the ROM of the fingers is observed at the latest follow-up.

The percentage of the total active motion of the fingers measured at the MP joint immediately after surgery and at 4 months after surgery is shown in Table 1. Numbness of the thumb, index, middle, and ring fingers, which persisted after the first operation, had improved significantly. He continued to have mild numbness in the index and ring fingers. On the Semmes-Weinstein test, the anesthesia of the index finger noted before surgery had improved to 3.84 (within the diminished protective sensation range of 3.84–4.31) at 4 months postoperatively. Complete ROM was regained in all the fingers, and no pain was perceived at follow-up (Figures 7(a–d)). He was able to use the three-finger pinch to lift a weight of 4.3 kg (normal average: 5 kg). His final grip strength was 18.8 kg (normal average: 50.3 kg).

**Table 1.** Total active motion of the metacarpophalangeal joints.

	Immediately after surgery	4 months postoperatively
Thumb	49%	86%
Index finger	37%	89%
Middle finger	27%	83%
Ring finger	17%	87%
Small finger	43%	95%

## Discussion

High-pressure injection injury is caused by an accidental injection by a paint gun or a pinhole in a hose (due to cracking). Injected substances include paint, thinner, metal, plastic, cement, water, oil, and grease. The fluid is injected at high pressure and penetrates deeply through the skin. These substances cause ischemia and tissue necrosis. The adipose tissue becomes necrosed after injury involving thinners [4], and it is often necessary to amputate the hand. Since the symptoms are mild in the early stage, there is often a delay in visiting the hospital. The risk of infection escalates, and treatment becomes more difficult as the time delay increases. Therefore, it is important to perform surgical treatment as early as possible [5,6].

The mechanism of high-pressure injection injuries postulated by Kaufman states that the subcutaneously injected fluid infiltrates the loose connective tissue along the fascia [7,8]. The injected fluid progresses along the digital neurovascular bundle, passes through the lumbrical space to the dorsal side, and progresses from the carpal tunnel to the Parona’s space of the forearm [1]. In the present case, the high-pressure injection injury occurred in the center of the palm, and the injected fluid seeped through the carpal tunnel proximally. It entered two layers in the thenar region, between the palmar aponeurosis and transverse carpal ligament. Distally, it tracked to all MP joints of the fingers, along the digital neurovascular bundles. Especially in the ring finger, it penetrated the dorsal MP joint through the lumbrical space. The fluid infiltrated an extensive area proximally and distally. Therefore, we could not completely debride it (Figure 5(c)). Once the necrotic tissue was debrided thoroughly, all flexor tendons and the median nerve were exposed. The paint could thus be removed sufficiently in the third operation. The patient’s neurological symptoms and ROM of the fingers improved significantly thereafter. However, there is no clarity regarding the early response and long-term progress in such cases.

The components of epoxy resin paint include a large number of carcinogens, such as toluene (18%); phenol, 4-dodecyl-, branched (9%); xylene (9%); 2-propanol (9%); ethylbenzene (9%); isobutanol (9%); solvent naphtha (petroleum), light arom (0.9%); and Stoddard solvent (0.9%) [9]. Epoxy resin paint has skin corrosive/irritant properties and reproductive toxicity. Remnants could cause cancer due to the presence of toluene and xylene [9]. The physical characteristics of epoxy resin paint include hardening at room temperature. Additionally, it has excellent water- and salt-resistant properties and is often used for painting ships and reinforcing cracks. The fluid cures in 12–24 h and volatilizes toxic substances when it hardens (volatile toxicity), which ends once curing ceases. There is an anticaking agent that is available, but it is tissue-toxic, and its application to tissue during surgery is prohibited [9]. Based on the above characteristics, the best treatment in such cases is to remove the paint before it hardens completely. Thus, we decided to perform a thorough debridement.

However, the paint did not harden during the first surgery, and the boundaries along the surrounding tissue were unclear. It adhered to the tissue like a chewing gum, which rendered its complete removal difficult. Despite this, the patient’s pain, sensory disturbance, and neuropathy decreased, and the ROM of the fingers improved after the first surgery. We believe that the pressure within the carpal tunnel decreased when the tunnel was opened, which led to improvements in the median neuropathy and ROM of the flexor tendons.

The paint had not cured, and the operation room was filled with the odors of xylene and toluene during the first surgery. All operating room staff were wearing surgical masks; however, these masks were unable to protect against the volatile toxicity of epoxy resin paint. If protective masks and goggles that can keep out volatile toxic gas are available, it is advisable that the surgical staff wear them. There was concern about the damage to the median and digital nerves due to the paint’s volatile toxicity. During the second surgery, the curing of the paint was not complete because it was performed less than 24 h after the injury. An irritating odor was noted during the operation, but some hardening of the paint was observed. The patient’s neurological symptoms further improved after this surgery. Since the volatile toxicity disappears once the paint is cured, it is possible that the soft tissue damage secondary to volatile toxicity had been alleviated after the second surgery. The paint releases toxic environmental hormones even after curing. The adhesion between the paint and tissue disappeared, so the excision was easier in the second surgery than in the first surgery.

From the above experience and based on the findings of previous studies [2,6,10], we believe that it is appropriate to perform repeated and thorough debridement before the defect is reconstructed using a flap. An early surgery may result in tension-free incision and prevent compartment syndrome that is caused by increased pressure and necrosis [11].

In the present case, a pedicled groin flap was used for reconstruction. A free skin graft and thin flap could cause tissue atrophy, leading to the adhesion of the flexor tendon and median nerve. This is detrimental to achieving tendon and nerve coverage. Therefore, we chose a pedicled groin flap because it had the advantage of preventing tissue scarring, tendon adhesion, and contracture of the fingers. Rehabilitation was restarted to accomplish an active ROM of the fingers. Since we chose a pedicled groin flap that could cover the flexor tendon and median nerve with sufficient thickness and volume, the palm was bulky after flap engraftment. However, the ROM of the fingers had improved to a near-normal level but was not 100%. We plan to perform a defatting surgery on the flap in the future.

The long-term course in such cases is unpredictable. There are a few reports of retained epoxy resin paint in the body. Such patients require follow-up for a long time. Steffen et al. [12] reported a case of multiple granulomatosis after subcutaneous injection of epoxy resin. We plan to follow-up our patient for the potential development of carcinomas, appearance of adhesions due to granulation, and delayed inflammation.

## Conclusion

We reported a case of widespread high-pressure injection injury caused by epoxy resin paint and described the short-term course and its management, as very few reports exist on this topic. The epoxy resin paint was extracted after it had hardened at room temperature. However, as the paint was potentially volatile during hardening, we considered it necessary to remove the paint as early as possible. To eliminate this trauma, factory equipment inspections, double-checking during the application, and high-pressure spray automation are required. In the unlikely event of such injuries, even if the wound is small, the patient should immediately go to a hospital and seek appropriate treatment. It is necessary to educate not only workers but also the managers of companies.
